# Functional Gene Expression Signatures from On-Treatment Tumor Specimens Predict Anti-PD1 Blockade Response in Metastatic Melanoma

**DOI:** 10.3390/biom13010058

**Published:** 2022-12-27

**Authors:** Shuzhao Chen, Limei Zhang, Haocheng Lin, Yang Liang, Yun Wang

**Affiliations:** 1 Department of Hematologic Oncology, State Key Laboratory of Oncology in South China, Sun Yat-sen University Cancer Center, Collaborative Innovation Center for Cancer Medicine, Guangzhou 510060, China; 2 Department of Oncology, The Eighth Affiliated Hospital of Sun Yat-sen University, Shenzhen 518033, China

**Keywords:** functional gene expression signatures, on-treatment tumor specimens, anti-PD1 blockade response, metastatic melanoma

## Abstract

Functional gene expression signatures (FGES) from pretreatment biopsy samples have been used to predict the responses of metastatic melanoma to immune checkpoint blockade (ICB) therapies. However, there are no predictive FGE signatures from patients receiving treatment. Here, using the Elastic Net Regression (ENLR) algorithm, we analyzed transcriptomic and matching clinical data from a dataset of patients with metastatic melanoma treated with ICB therapies and produced an FGE signature for pretreatment (FGES-PRE) and on-treatment (FGES-ON). Both the FGES-PRE and FGES-ON signatures are validated in three independent datasets of metastatic melanoma as the validation set, achieving area under the curve (AUC) values of 0.44–0.81 and 0.82–0.83, respectively. Then, we combined all test samples and obtained AUCs of 0.71 and 0.82 for the FGES-PRE and FGES-ON signatures, respectively. The FGES-ON signatures had a higher predictive value for prognosis than the FGES-PRE signatures. The FGES-PRE and FGES-ON signatures were divided into high- and low-risk scores using the signature score mean value. Patients with a high FGE signature score had better survival outcomes than those with low scores. Overall, we determined that the FGES-ON signature is an effective biomarker for metastatic melanoma patients receiving ICB therapy. This work would provide an important theoretical basis for applying FGE signatures derived from on-treatment tumor samples to predict patients’ therapeutic response to ICB therapies.

## 1. Introduction

Immune checkpoint blockade (ICB) therapies are a powerful strategy for treating metastatic melanoma and other cancer types; however, most patients do not respond or achieve complete clinical responses [1,2]. Overusing and misusing drugs can lead to increased side effects, drug resistance, and increased costs [3]. Therefore, predictive biomarkers are necessary to determine which patients will benefit most from ICB therapy.

Previous studies have identified predictive biomarkers for ICB treatment response in metastatic melanoma, including the tumor mutational burden, eosinophilic count, shelterin complex expression, glycolytic activity, and aneuploidy [4,5,6,7,8]. However, these signatures are based on preclinical models and clinical cohorts using pretreatment biopsies.

Moreover, because of the lack of reproducibility and batch effect, there are marked differences between the actual and predicted responses to ICB therapy using these pretreatment signatures, implying inaccurate predictive ability.

For example, Xiao et al. identified that ImmuneCell.Sig did not show robust prediction performance across four melanoma datasets [9,10]. Additionally, Carter et al. concluded that there is currently insufficient evidence to consider the immune-predictive scores (IMPRES) as a reliable predictor of ICB response in melanoma [11,12].

The transcriptomic analysis offers an opportunity to understand the complex cellular heterogeneity in tumors and to discover predictive biomarkers for ICB therapy [13,14]. The tumor microenvironment (TME) plays a crucial role in patient survival and response to therapy [15,16]. Combining gene expression signatures or tumoral pathways with an understanding of the TME-associated immune cells may provide a better predictive model for ICB therapy. For example, a recent study combined different functional gene expression (FGE) signatures representing the major functional components, such as immune, stromal, and other cell populations in the tumor. This study identified a favorable immunotherapy response in patients with metastatic melanoma [17]. Another study developed pathway-related signatures to predict the response to anti-PD1-based therapies [18]. However, these studies contained too many gene expression patterns or pathway-related genes and were too complex to be applied practically. Therefore, another clinically focused prediction signature is needed for predicting patient response to ICB therapies.

In this study, we developed FGE-based signatures to predict the response to ICB therapies in metastatic melanoma using transcriptome data and clinical information from four databases for both pre- and on-treatment tumor specimens.

## 2. Methods

### 2.1. Studies and Patient Selection

We analyzed four ICB response datasets: the Riaz et al. dataset (available in GEO: GSE120575), Gide et al. (available in BioProject: PRJEB23709), MGH cohort (available in GEO: GSE115821 and GSE168204), and Abril-Rodriguez et al. (available in dbGaP: phs001919.v1.p1). Patients were excluded if they (1) lacked RNA sequencing data and (2) did not have a response evaluation. Patient responses to the ICB were defined according to the RECIST criteria (https://recist.eortc.org accessed on 26 November 2022) [19]. Patients were divided into responders and nonresponders. Responders(R): patients with a complete response (CR), partial response (PR), or stable disease (SD) with progression-free survival (PFS) longer than 180 days. Nonresponders (NR): patients with SD with PFS shorter than 180 days and progression disease (PD).

### 2.2. Signature Score Calculation

The ssGSEA was used to estimate the scores of 30 functional gene signatures using the “GSVA” package. An elastic net penalized logistic regression model was implemented to identify FGE signatures associated with the response to ICB therapy using the “glmnet” R package [20]. To avoid overfitting, we performed a three-fold cross-validation of the training dataset. A cost-sensitive algorithm was used to address imbalanced classification. A receiver operating characteristic (ROC) curve was plotted to evaluate prediction performance.

ICB prediction accuracy of the FGE signature scores was quantified with the area under the curve (AUC) calculated using the R software “ROCR” package. The Youden index method was used to select the optimal threshold point from the ROC curve in the Riaz et al. dataset [21,22]. After combining all the test samples, we used the Matthews correlation coefficient to assess the correlation between the predicted and the response outcome [23]. We also calculated the odds ratio of each sample based on the signature score.

### 2.3. Statistical Analysis

The significance of the differences between responders and nonresponders in the ICB cohorts was tested using a Wilcoxon one-sided rank sum test.

Survival analyses were performed using Kaplan–Meier (KM) estimates of survival probability and log-rank tests. The cutoff point was the mean odds ratio, and samples were separated into a low-signature score group and a high-signature score group. The proportional hazards assumption was held for all models, and hazard ratios (HR) were calculated using the Cox regression analysis. The proportional hazard assumption of the Cox model in all cohorts was displayed graphically (Figures S5 and S6). Multi-Cox regression analyses were performed using the survival R package. All statistical analyses were performed, and data were plotted using R v. 4.2.1. and GraphPad Prism v. 8.0 (https://www.graphpad.com/scientific-software/prism/ accessed on 26 November 2022).

## 3. Results

### 3.1. Patient Characteristics

In this study, we searched and analyzed four available human datasets with RNA-seq data and clinical information from metastatic melanoma patients treated with anti-PD-1, anti-PD-L1, anti-CTLA-4 monotherapy, or a combination of any two.

**Figure 1:**
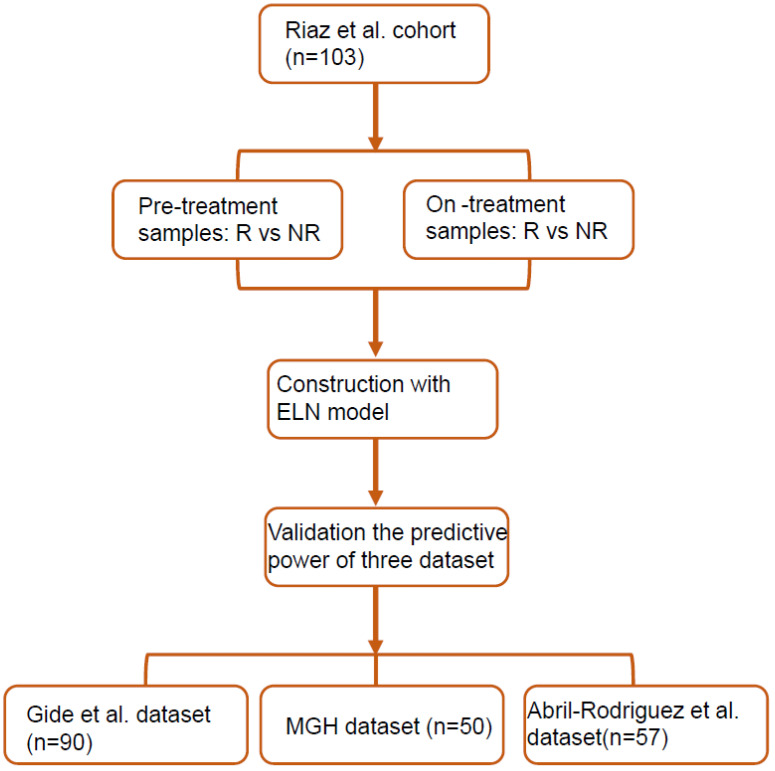
The flow chart of FGE signature score construction.

Since the sample size of the Riaz et al. dataset is the largest among all four datasets [24], it was used as the training set, and the samples from the Gide et al., MGH, and Abril-Rodriguez et al. datasets were used as the validation set [18,25,26]. The construction and validation of the elastic net regression (ELNR) model are illustrated in Figure 1.

### 3.2. FGE-Based Signature for Pretreatment Samples

**Figure 2:**
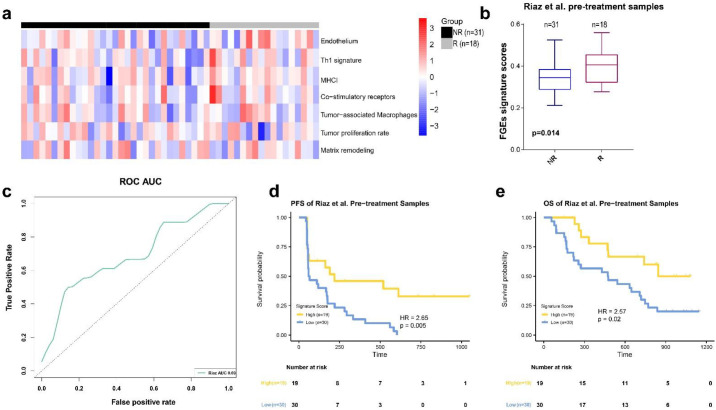
FGE-based signature for pretreatment samples (FGE-PRE). (**a**) Heatmap representing the single sample gene set enrichment analysis value of pretreatment nonresponders (NR) and responders (R) in the Riaz et al. cohort. (**b**) Boxplot of FGE-PRE signature scores for pretreatment samples from the Riaz et al. datasets. *p* values were computed via a one-sided Wilcoxon rank-sum test. (**c**) Receiver operating correlation curve and area under the curve of FGE-PRE signatures for pretreatment samples from the Riaz et al. cohort. (**d**,**e**) Kaplan–Meier curves of PFS and overall survival for pretreatment samples based on FGE-PRE signature scores for the Riaz et al. cohort.

We selected 30 FGE signatures representing the major functional components and stromal, immune, and other cellular populations [6,17]. First, we calculated each signature’s scores for each sample in the Riaz et al. training set by the ssGSEA algorithm. Next, the ENLR model was used to establish predictive FGE-based signatures based on pretreatment samples in the Riaz et al. training set (Figure 1). For the Riaz et al. dataset, a cross-validation procedure and cost-sensitivity method were implemented to determine the optimized penalty parameter with the error to calculate the effect size of each candidate FGE signature (Figure S1a,b). Finally, seven hub FGE signatures were defined as the most effective variables for predicting the response to ICB therapy, including the endothelium, Th1 signature, MHC I, costimulatory receptors, tumor-associated macrophages, tumor proliferation rate, and matrix remodeling (Figure 2a and Figure S1c).

The Th1 signature, MHC I, and costimulatory receptors are related to the anti-TME activity, whereas the endothelium and matrix remodeling are related to malignant cell properties. The tumor proliferation rate is related to angiogenesis and fibrosis. Furthermore, we used the effective sizes as weights, calculated the weighted average of ssGSEA scores of these seven FGE signatures, and named it the FGE-based signature score (FGES). 

We found that the FGES of pretreatment samples (FGES-PRE) from patients who responded to ICB was higher than those with no response in the Riaz et al. cohort (*p* = 0.014, Figure 2b). The AUC of the ROC curve was 0.69 (Figure 2c).

Next, patients in the training cohort were divided into high- and low-risk groups using the mean of their odds ratios derived from FGES-PRE scores. KM survival analysis was performed for PFS and overall survival (OS). We found that patients in the high-score group showed a significantly better PFS (HR = 2.65, *p* = 0.005, Figure 2d) and OS (HR = 2.57, *p* = 0.02, Figure 2e) compared to those in the low-score group. The response rate in the high-score group was higher than in the low-score group (Figure S3a).

**Figure 3:**
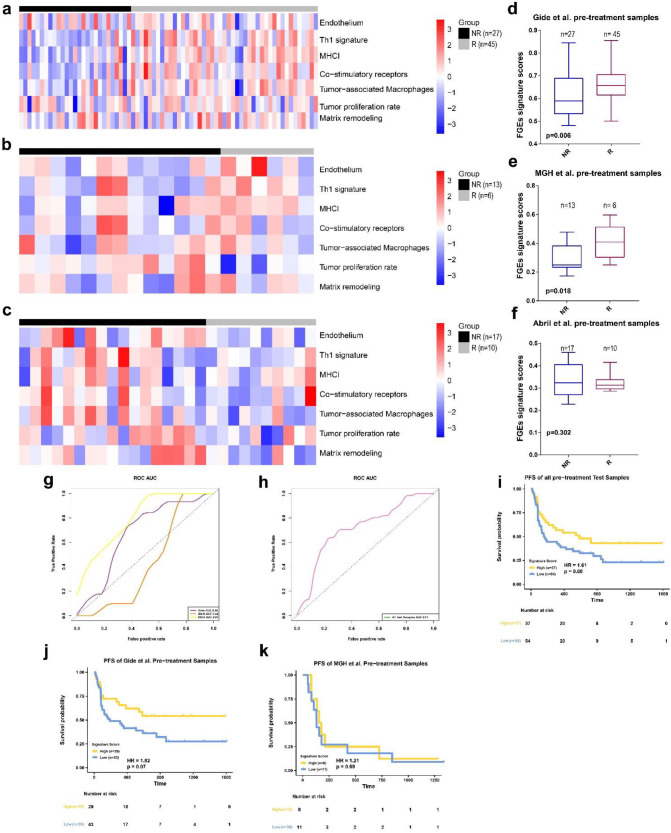
FGE-based signature for pretreatment samples (FGE-PRE) (**a**–**c**) Heatmap representing the single sample gene set enrichment analysis value of pretreatment nonresponders (NR) and responders (R) in the Gide et al., MGH, and Abril-Rodriguez et al. cohorts. (**d**–**f**) Boxplot of FGE-PRE signature scores for pretreatment samples from the Gide et al., MGH, and Abril-Rodriguez et al. cohorts. *p* values were computed via a one-sided Wilcoxon rank-sum test. (**g**) Receiver operating correlation curve and area under the curve of FGE-PRE signatures for pretreatment samples from the Gide et al., MGH, and Abril-Rodriguez et al. cohorts. (**h**) Receiver operating correlation curve and area under the curve of FGE-PRE signatures for pretreatment samples from all test samples combined. (**i**–**k**) Kaplan–Meier curves of PFS for pretreatment samples based on FGE-PRE signature scores for all test samples, Gide et al., and MGH cohorts.

In order to validate these results, a similar analysis was performed on the three independent datasets using the RNA-seq data from pretreatment samples (Figure 3a–c). The FGES-PRE score and odds ratio were calculated for each sample. The results of the Mann–Whitney U test showed that the FGES-PRE scores of R in the Gide et al. and MGH datasets were significantly higher than those of NR (Figure 3d,e). However, there was no significant difference between NR and R in the Abril-Rodriguez et al. dataset (Figure 3f). Similarly, we measured the ROC curves and calculated AUCs of 0.68, 0.81, and 0.44 for Gide et al., MGH, and Abril-Rodriguez et al., respectively (Figure 3g). All tested pretreatment samples of the independent datasets yield an AUC value of 0.70 (Figure 3h). Using the Youden index [22], we found that the optimal value for the Riaz et al. dataset was 0.419. The prediction accuracy of all tested pre-samples for predicting the treatment response was 0.62. The Matthew correlation coefficient was −0.01. 

Subsequently, subjects were divided into high- and low-risk score groups using the mean value of each sample’s odd ratios derived from the FGES-PRE score. We then performed KM analyses for PFS and OS for each individual and for all test samples. Patients with high FGES-PRE scores had significantly improved PFS (HR = 1.61, *p* = 0.08, Figure 3i) and OS (HR = 2.145, *p* = 0.03, Figure S3e) compared to those with low FGES-PRE scores in the combined cohort. To verify these results, we performed a similar analysis in the MGH and Gide et al. datasets (The samples of the Abril-Rodriguez et al. dataset did not have survival information). In the Gide et al. dataset, PFS (HR = 1.82, *p* = 0.07, Figure 3j) but not OS (HR = 1.58, *p* = 0.25, Figure S3f) was significantly longer in patients with high FGES-PRE scores than in those with low FGES-PRE scores. Analysis of the MGH dataset showed that high signature scores significantly improved OS (HR = 5.26, *p* = 0.02, Figure S3g) but not PFS (HR = 1.21, *p* = 0.69, Figure 3k) compared with those with a low score. Furthermore, we found that the response rate of the high-score group in the Gide et al. and MGH cohorts were higher than those of the low-score group (Figure S3b,c); however, contrary results were observed for the Abril-Rodriguez et al. cohort (Figure S3d).

To determine whether the FGE signature was an independent prognostic covariate, we tested it in multivariable Cox analyses in Riaz et al. cohort, which had extra clinical covariates. The FGE signature was an independent covariate for OS and PFS survival in the Riaz et al. cohort after adjusting for additional factors (Figure S3h,i).

Collectively, these results revealed that the FGES-PRE signature derived from pretreatment samples did not have a robust or consistent ability for predicting the response and survival of metastatic melanoma patients receiving ICB therapies.

### 3.3. FGE-Based Signature for On-Treatment Samples

**Figure 4:**
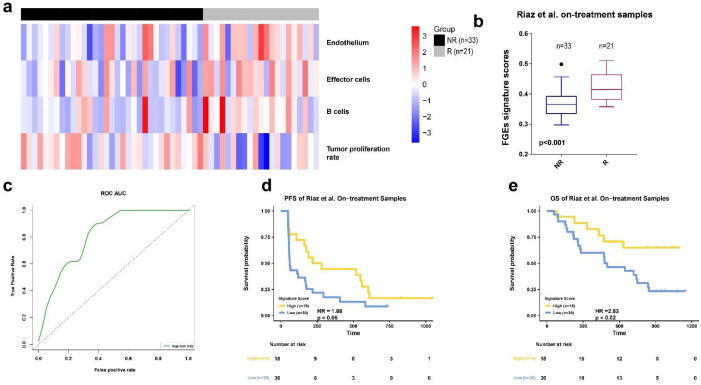
FGE-based signature for on-treatment samples (FGE-ON) (**a**) Heatmap representing the single sample gene set enrichment analysis value of on-treatment nonresponders (NR) and responders (R) in the Riaz et al. cohort. (**b**) Boxplot of FGE-ON signature scores for on-treatment samples from the Riaz et al. datasets. p values were computed via a one-sided Wilcoxon rank-sum test. (**c**) Receiver operating correlation curve and area under the curve of FGE-ON signatures for on-treatment samples from the Riaz et al. cohort. (**d**,**e**) Kaplan–Meier curves of PFS and OS for on-treatment samples based on FGE-ON signature scores for the Riaz et al. cohort.

Next, the predictive performance of the FGE signatures derived from the on-treatment samples was investigated using the four datasets. We calculated each signatures scores for each sample in Riaz et al. training set by ssGSEA algorithm. Then, we used the ENLR model to identify the FGE signatures with the highest predictive accuracy (Figure S2a,b). We identified four gene signatures for the endothelium, effector cells, B cells, and tumor proliferation rate. Both effector and B cells are related to anti-TME effects (Figure 4a and Figure S2c). Furthermore, we used the effective sizes as weights and calculated the weighted average of the ssGSEA scores of these four FGE signatures and named it the FGE-based signature for the on-treatment sample (FGES-ON) scores. We also performed a Mann–Whitney U test on the FGES-ON scores and found that Rs were higher than NRs in Riaz et al. dataset (Figure 4b, *p* < 0.001). The association between signature scores and the overall survival time was also evaluated by Kaplan–Meier analysis. The AUC was 0.82 for the Riaz et al. cohort. We also observed that patients with high scores had significantly longer PFS and OS than those with low scores (Figure 4d,e). 

**Figure 5:**
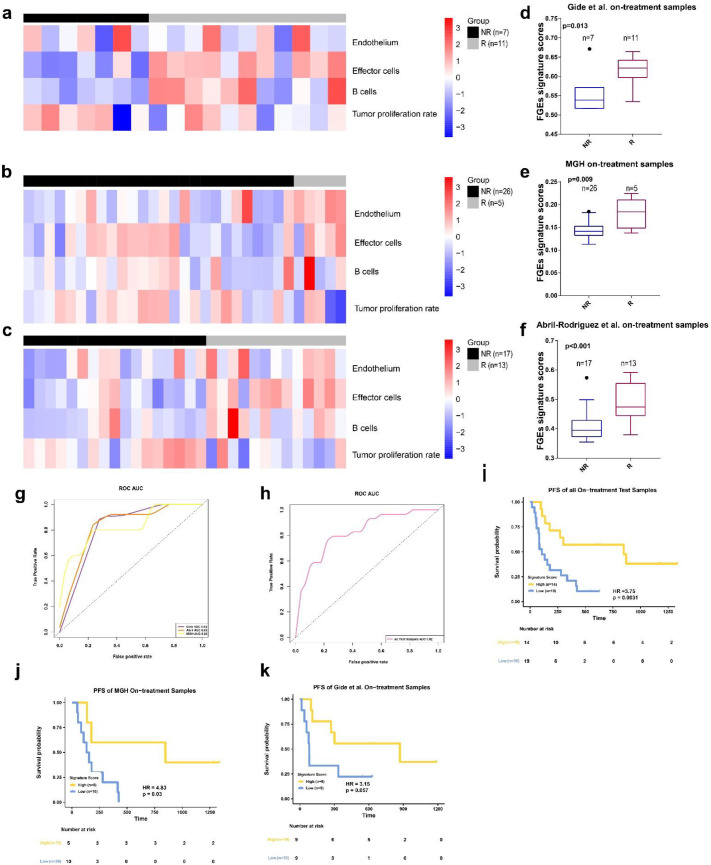
FGE-based signature for on-treatment samples (FGE-ON) (**a**–**c**) Heatmap representing the single sample gene set enrichment analysis value of on-treatment nonresponders (NR) and responders (R) in the Gide et al., MGH, and Abril-Rodriguez et al. cohorts. (**d**–**f**) Boxplot of FGE-ON signature scores for on-treatment samples from Gide et al., MGH, and Abril-Rodriguez et al. cohorts. *p* values were computed via a one-sided Wilcoxon rank-sum test. (**g**) Receiver operating correlation curve and area under the curve of FGE-ON signatures for on-treatment samples from the Gide et al., MGH, and Abril-Rodriguez et al. cohorts. (**h**) Receiver operating correlation curve and area under the curve of FGE-ON signatures for on-treatment samples from all test samples, which combined the Gide et al., MGH, and Abril-Rodriguez et al. cohorts. (**i**–**k**) Kaplan–Meier curves of PFS for on-treatment samples based on FGE-ON signature scores for all test samples, Gide et al. and MGH cohorts.

We discovered that the signature scores of Rs were significantly higher than those of NRs in all three cohorts (Figure 5d–f).

The AUCs were 0.82, 0.83, and 0.83 for the Gide et al., MGH et al., and Abril-Rodriguez et al. datasets, respectively (Figure 5g). The combined on-treatment samples of the three datasets yielded an AUC value of 0.82 (Figure 5h). Using the Youden index, we found that the optimal value for the Riaz et al. dataset was 0.377. The prediction accuracy of the combined on-treatment samples for predicting the treatment response was 0.70. The Matthew correlation coefficient was 0.41. 

Patients with high FGES-ON scores had significantly improved PFS and OS compared with low scores in all test samples (HR = 3.75, *p* = 0.0031, Figure 5i; HR = 3.50, *p* = 0.016, Figure S4e). The response rate in high score group was higher than low score group (Figure S4a). To verify these results, we also performed a similar analysis in the Gide et al. and MGH datasets (The samples of Abril-Rodriguez et al. dataset do not have survival information). 

In both datasets, PFS was significantly longer for patients with high FGES-ON scores than for those with low scores (For Gide et al: HR = 3.15, *p* = 0.057, Figure 5k; For MGH: HR = 4.83, *p* = 0.03, Figure 5j). High signature scores featured significantly improved OS (HR = 3.84, *p* = 0.07, Figure S4g) in the MGH dataset but not in the Gide et al. dataset (HR = 3.37, *p* = 0.15, Figure S4f) compared to those with low signature scores.

Furthermore, we found that the response rate of high score group in the Gide et al. MGH cohorts and Abril-Rodriguez et al. cohort were higher than those of low score group (Figure S4b–d).

To determine whether the FGES-ON was an independent prognostic covariate, we tested it in multivariable Cox analyses in the Riaz et al. cohort, which had extra clinical covariates. The FGES-ON was an independent covariate for PFS and OS survival in the Riaz et al. cohort after adjusting for additional factors (Figure S4h,i).

Based on the results of on-treatment samples analysis, we concluded that FGES-ON is more consistent and robust than FGES-PRE in predicting patients’ clinical responses to ICB therapies. In addition, compared with the FGES-PRE scores, the FGES-ON scores could better stratify patients into groups of better and worse prognoses.

## 4. Discussion

ICB therapy can only produce a durable response in a subset of patients with metastatic melanoma [1,2]. Therefore, identifying and using robust biomarkers to determine the best candidates for ICB therapy is critical. Most studies have identified gene signatures that show high predictive power for ICB therapy response in patients with metastatic melanoma. However, most of the signatures were derived from pretreatment samples, and none of them focused on the TME-associated immune cells. In this study, we analyzed both pre- and on-treatment tumor specimens in four independent cohorts and developed functional gene expression signatures regarding tumor and immune components to predict ICB response in patients with metastatic melanoma. We demonstrated that FGE signatures derived from on-treatment could predict responsiveness to ICB and were strongly associated with increased PFS.

Previous studies indicated that on-treatment tumor samples could reliably predict patient endocrine therapy responses compared to pretreatment samples in breast cancer [27,28,29]. However, thus far, there has been no information on whether functional gene signatures of on-treatment samples can predict patient responses to ICB therapies and outcomes in metastatic melanoma.

In this study, we constructed FGE signatures derived from on-treatment samples using RNA-seq data from four independent cohorts to predict the clinical response and survival of patients with metastatic melanoma. Our data indicate that clinicians should evaluate the signatures from both pretreatment and ICB-treated patients.

In addition, our findings may have implications for clinical management because most tumor specimens were obtained before ICB treatment. Accurate FGES-ON scores can be used to select patients who are likely to benefit from ICB treatment. In contrast, identifying ICB therapy nonresponsiveness earlier would enable better initial treatment selection, such as tumor-targeted therapy. This would reduce the disease burden and side effects and lead to better disease outcomes for patients.

We identified endothelium, effector cells, B cells, and the tumor proliferation rate as FGE signatures in the present study. Moreover, we used these to construct a predictive model using analysis of on-treatment samples. Interestingly, the endothelial component was also identified. Recent research identified that tumor-associated high endothelial cells (TA-HECs) play a vital role in migrating peripheral lymphocytes to tumor tissues and increasing the abundance of tumor-infiltrating T cells [30]. They further showed that increasing TA-HEC density and maturation in the TME is associated with a better anti-PD1/anti-CTLA-4 treatment response and survival rate in patients with metastatic melanoma [30]. Vascular endothelial cells can also inhibit immune cell function in the TME by expressing PD-L1 [31,32]. In addition, high PD-L1 expression in cancer cells is associated with a favorable prognosis and better disease-free survival in response to anti-PD-1 inhibitors [33,34]. Together, these studies provide evidence of the important role of the endothelium component in conferring a response to ICB therapies. This information correlates with our results and provides information on how the endothelium component can predict ICB therapy response.

Notably, the FGES-ON signature also includes a B-cell signature. A previous study identified a B lineage signature correlated with improved survival of sarcoma patients receiving anti-PD-1 therapy [35,36]. Another study suggested that patients with NSCLC and increased plasma cell signatures treated with anti-PD-1/anti-PD-L1 therapy displayed good survival, regardless of CD8^+^ T-cell infiltration [37]. Our results further highlight the role of B cells in immunotherapy responses, especially in the on-treatment samples.

Combination therapy between immunotherapy and local therapies, such as chemotherapy, radiation therapy, and targeted therapy, is gaining more and more attention among researchers in clinical practice [38,39]. For example, several clinical trials were launched to investigate the potential synergistic effect of immunotherapy and radiotherapy in melanoma patients [38]. The combination strategy with a synergistic anti-tumor activity using immunotherapy as a partner of local therapies represents a promising field for the treatment of patients with melanoma. With increasing research in developing robust biomarkers to predict the efficacy of therapeutic modalities and guide clinical decision-making, combination therapy among ICBs, radiation therapy, adoptive cell therapy, cancer vaccines, and small molecule inhibitors are expected. The accuracy of the predictive ability of the FGES in combination therapy needs to be further verified. In this regard, a truly patient-centered, individualized approach is what cancer immunotherapy needs to succeed in the future.

In summary, using the ENLR algorithm, we discovered four FGE signatures derived from on-treatment samples and produced an FGES-ON signature that consistently and accurately predicts patient response to ICB therapy. This signature not only predicts the prognosis response to ICB therapy but also provides insight into clinical decision-making in managing patients with metastatic melanoma. Prospective studies with larger sample sizes from multiple centers are necessary to validate these signatures further.

## Data Availability

The datasets presented in this study can be found in online repositories. The names of the accession number(s) can be found in the article.

## Supplementary Materials

The following are available online at https://www.mdpi.com/article/10.3390/biom13010058/s1, Figure S1: FGE-based signature for pre-treatment samples. Figure S2: FGE-based signature for on-treatment samples. Figure S3: FGE-based signature for pre-treatment samples. Figure S4: FGE-based signature for on-treatment samples. Figure S5: Graphical assessment of the proportional hazards assumption of the FGE-PRE signature in pre-treatment samples. Figure S6: Graphical assessment of the proportional hazards assumption of the FGE-ON signature in the on-treatment samples.
