# Supplementary material for: Functional Gene Expression Signatures from On-Treatment Tumor Specimens Predict Anti-PD1 Blockade Response in Metastatic Melanoma
Source: Biomolecules. 2022 Dec 27;13(1):58. doi: 10.3390/biom13010058 (PMC9855743; doi:10.3390/biom13010058)
Supplement: Supplementary file 1 [file biomolecules-13-00058-s001.zip › biomolecules-2090523-supplementary.pdf]

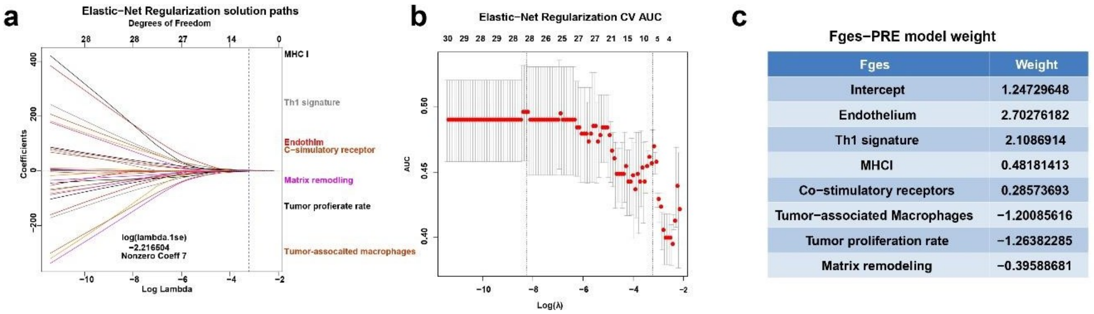

Figure S1. FGE-based signature for pre-treatment samples. (a, b). The model's training parameter selection process was used to generate the Riaz et al. pre-treatment samples to generate the FGE-PRE signature. To avoid overfitting, 3-fold cross-validation was performed with the parameter setting as "type.measure = auc, family = 'binomial'." (c). FGE-based signatures (FGE-PRE) consisted of seven selected frames associated with the effect sizes (variable weights) from the elastic net penalized logistic regression model.

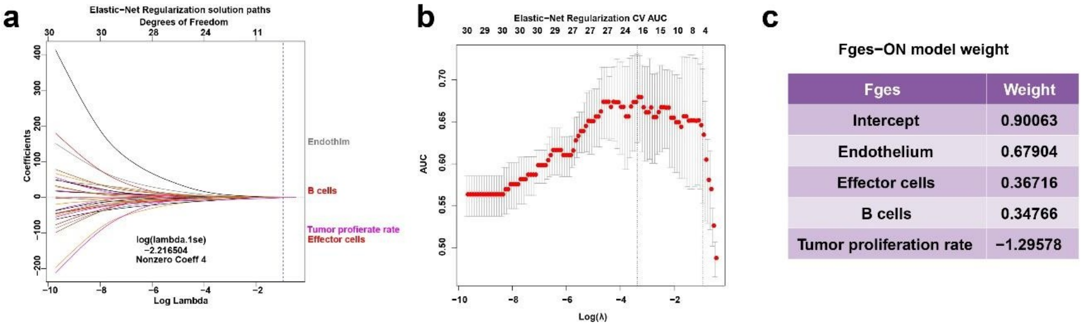

Figure S2. FGE-based signature for on-treatment samples. (a, b).The model's training parameter selection process to generate Riaz et al. on-treatment samples for the FGE-ON signature. To avoid overfitting, 3-fold cross-validation was performed with the parameter setting as "type.measure = auc, family = 'binomial'." (c). FGE-based signatures (FGE-ON) consists of four selected FGEs associated with the effect sizes (variable weights) from elastic net penalized logistic regression model.

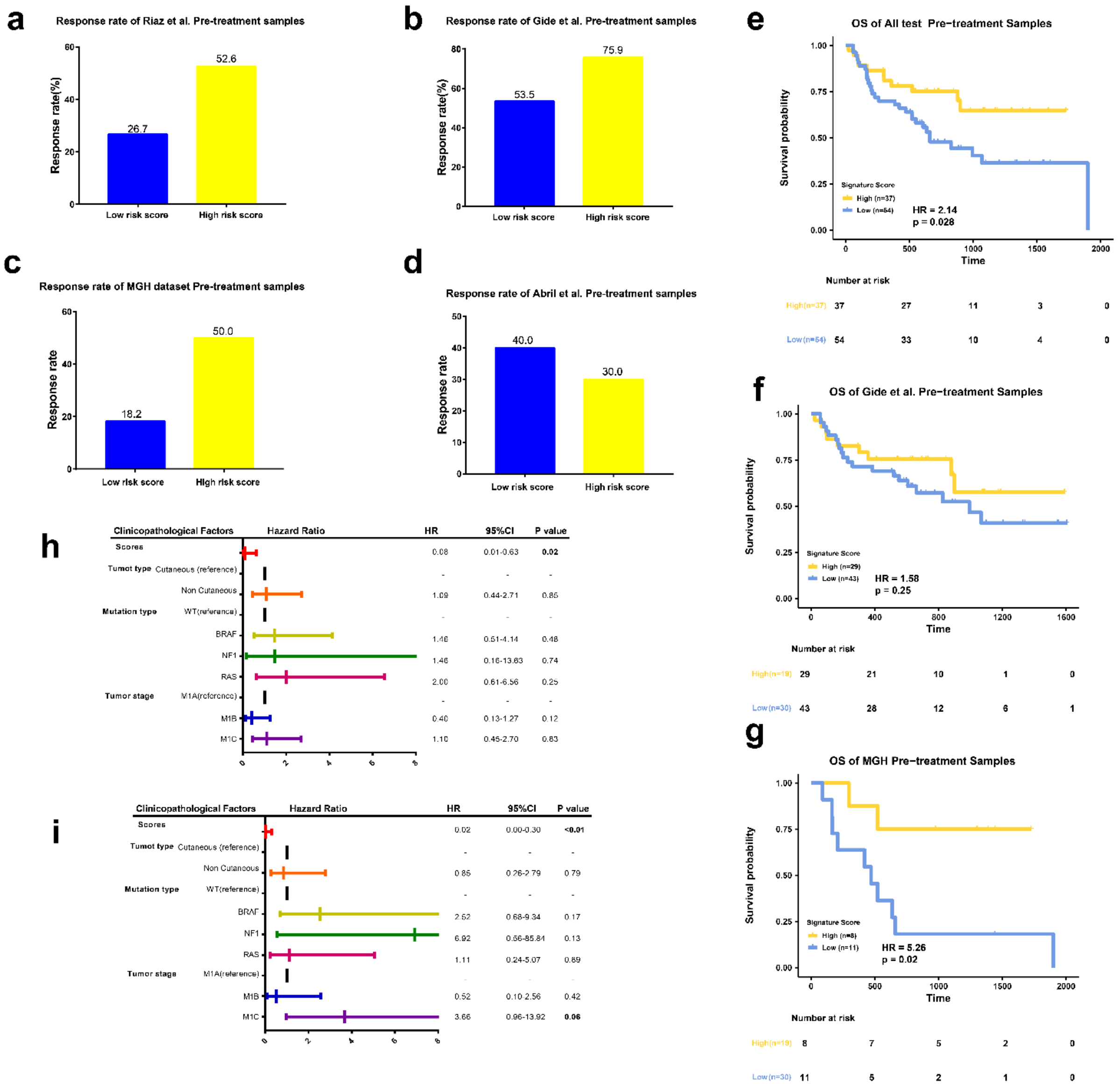

Figure S3. FGE-based signature for pre-treatment samples. (a-d) The response rate of pre-treatment samples between high and low signature scores groups. (e) Overall survival analysis of all tested pre-treatment samples. (f) Overall survival analysis of the Gide et al. pre-treatment samples. (g) Overall survival analysis of the MGH pre-treatment samples. (h-i) Multi-variate regression analysis of PFS(h) and OS(i) in pre-treatment samples patients from Riaz et al. cohort.

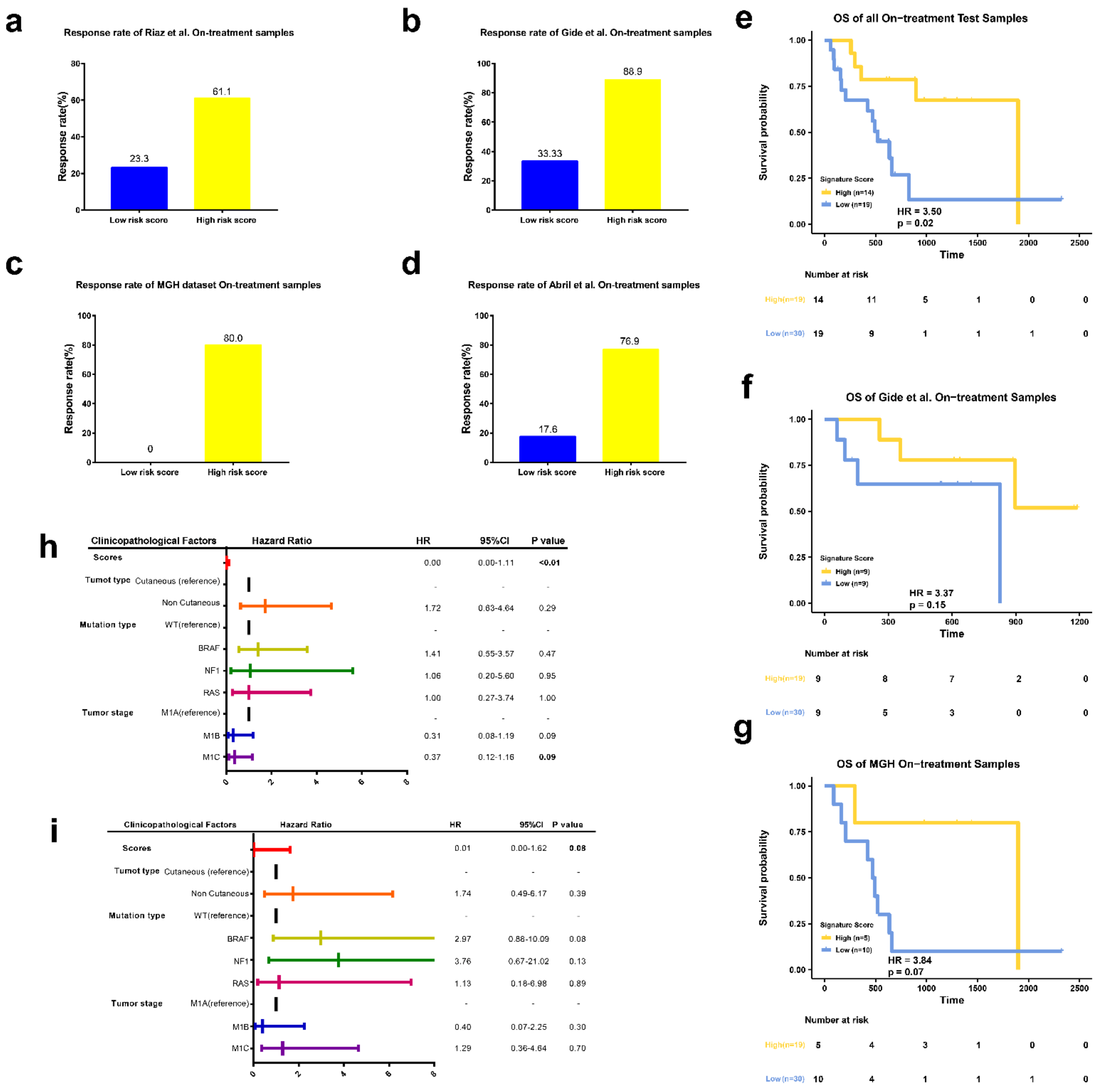

Figure S4. FGE-based signature for on-treatment samples. (a-d) The response rate of on-treatment samples between high and low signature scores groups. (e) Overall survival analysis of all tested on-treatment samples. (f) Overall survival analysis of the Gide et al. on-treatment samples. (g) Overall survival analysis of the MGH on-treatment samples. (h-i) Multi-variate regression analysis of PFS(h) and OS(i) in on-treatment samples patients from Riaz et al. cohort.

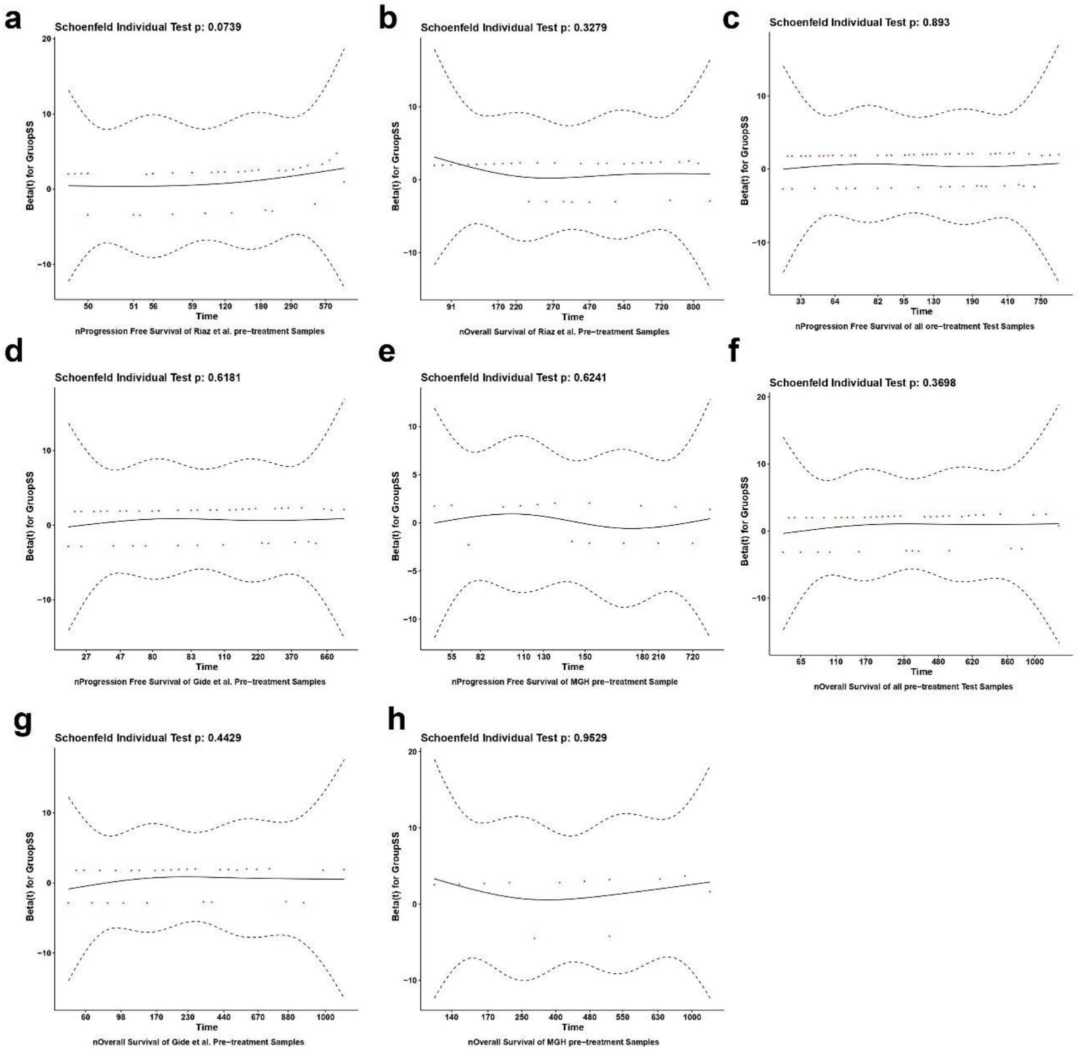

Figure S5. Graphical assessment of the proportional hazards assumption of the FGE-PRE signature in pre-treatment samples.

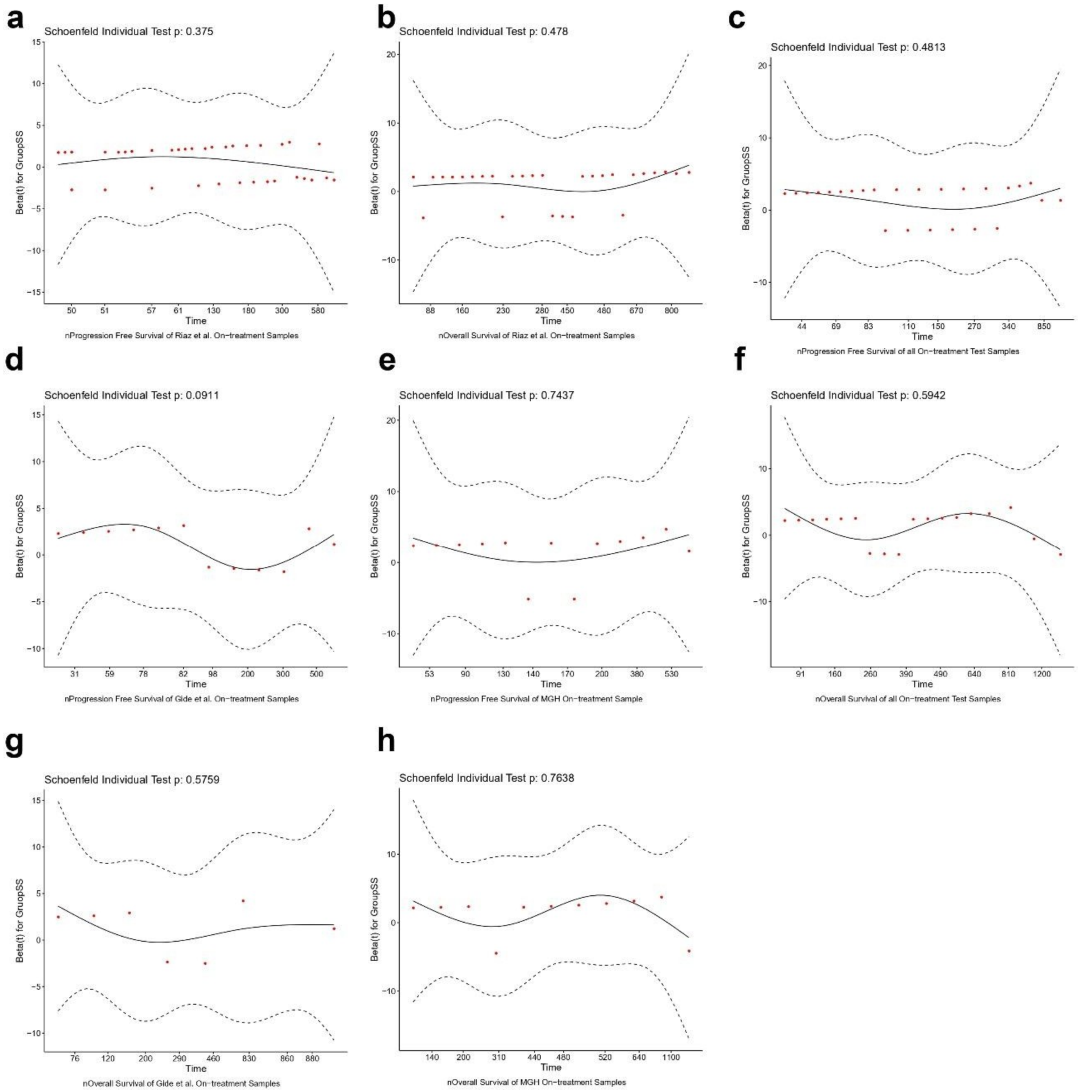

Figure S6. Graphical assessment of the proportional hazards assumption of the FGE-ON signature in the on-treatment samples.
